# Glomerulonephritis and Septic Pulmonary Embolism: A Rare but Life-Threatening Complication of Permanent Pacemaker Implantation

**DOI:** 10.7759/cureus.58196

**Published:** 2024-04-13

**Authors:** Yogesh S, Selva Krishna R, Suriya Prakash Srinivasan, Hariharan C, Karthigeyan TS, Sivakumar T, Gokulakrishnan H, Bala Manikandan, Sandhiya N, Gautam K

**Affiliations:** 1 Internal Medicine, Madras Medical College and Rajiv Gandhi Government General Hospital, Chennai, IND; 2 Internal Medicine, Madras Medical College and Rajiv Gandhi Government Medical College, Chennai, IND

**Keywords:** infection-related glomerulonephritis, infective endocarditis-associated glomerulonephritis, right-sided infective endocarditis, cardiac implant-associated infective endocarditis, cardiac device-related infective endocarditis, glomerulonephritis (gn), post-infectious glomerulonephritis

## Abstract

Infection-related glomerulonephritis (IRGN) is an immunologically mediated glomerular injury triggered by an extrarenal infection. Infective endocarditis-associated glomerular nephritis is an entity caused by infection of the cardiac valves. IRGN is most common in children, and post-streptococcal glomerulonephritis (PSGN) is commonest in the age group of 2-14 years. In contrast to childhood PSGN and epidemic PSGN, which usually resolve completely with antibiotics, IRGN in adults has a guarded prognosis. Cardiovascular implantable electronic device-associated infective endocarditis (CIED-IE) is a phenomenon for which the incidence is on the rise (0.1-5.1%). The most frequent CIED-IE pathogens were staphylococci or other Gram-positive bacteria. CIED-IE poses difficult management problems for the clinician. We present the case of a 50-year-old patient with a pacemaker who was found to have infective endocarditis and septic embolism.

## Introduction

Infection-related glomerulonephritis (IRGN) is a glomerular injury caused by the immune response triggered by an infection. Renal complications associated with infective endocarditis (IE) have been well-documented for over a century, with initial reports of glomerular damage dating back more than a hundred years [1-3]. Although it was initially believed to be primarily linked to embolism, subsequent research revealed that more than 80% of cases involved focal, segmental, or diffuse proliferative glomerulonephritis (GN), characterized by significant endocapillary proliferation and occasional infiltration of white blood cells. The challenges in making a definitive diagnosis are primarily due to the need to confirm the presence of the infection through culture and the identification of the infection site, typically through echocardiography.

In recent times, an emerging medical concern is cardiovascular implantable electronic device-associated infective endocarditis (CIED-IE), as these devices like implantable defibrillators, resynchronization devices, and permanent pacemakers are being more commonly used. This discussion focuses on a case of CIED-IE that presented as IRGN.

## Case presentation

We present the case of a 50-year-old patient who presented to the emergency department with complaints of low-grade fevers, shortness of breath, worsening edema, and hematuria. The patient was experiencing chest tightness and dyspnea on exertion, which gradually worsened in the last three to four weeks. His past history was significant for sick sinus syndrome in 2003 when he was managed with a permanent pacemaker insertion (VVIR mode). On follow-up, the pacemaker generator was replaced in 2016 in view of end of life. Two months later, he was found to have a collection over the pacemaker site, for which the pacemaker was re-implanted on the right infraclavicular area and post-op angiography was done which revealed 50% occlusion in the mid-left anterior descending (LAD) artery. Unfortunately, the patient didn't follow up as advised but continued to take his medications as recommended.

On examination, he was febrile and had pallor and pedal edema with a pulse rate of 80/min, a blood pressure of 110/70 mmHg, and an oxygen saturation of 98% in room air. His blood investigations are as shown in the table (Table 1). Urinalysis revealed red blood cell (RBC) casts and no significant proteinuria. Fundoscopy revealed Roth spots in both eyes. Electrocardiogram revealed asymmetrical T inversion in II, III, aVF, and V3-V6, intermittent pacing complexes with left bundle branch block (LBBB) morphology, and right ventricular (RV) apical pacing with a pacing rate of 70/min.

**Table 1:** Laboratory investigations

Parameters	Day 1 of admission	Day 4 of admission	Day 8 of admission
Hemoglobin (g/dL)	10.1	8.4	7.9
Total counts (cu.mm)	6100	4600	3600
Platelets (per microliter of blood)	95000	80000	79000
Urea (mg/dL)	94	136	159
Creatinine (mg/dL)	3.8	4.2	4.3
Sodium (mEq/L)	129	130	129
Potassium (mmol/L)	4.4	5.3	5.7

**Figure 1:**
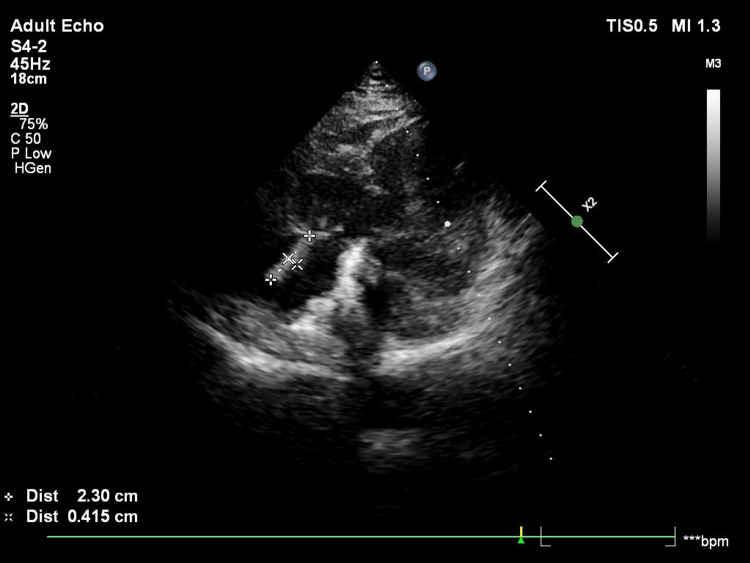
Echocardiogram revealing vegetation in the tricuspid leaflet

**Figure 2:**
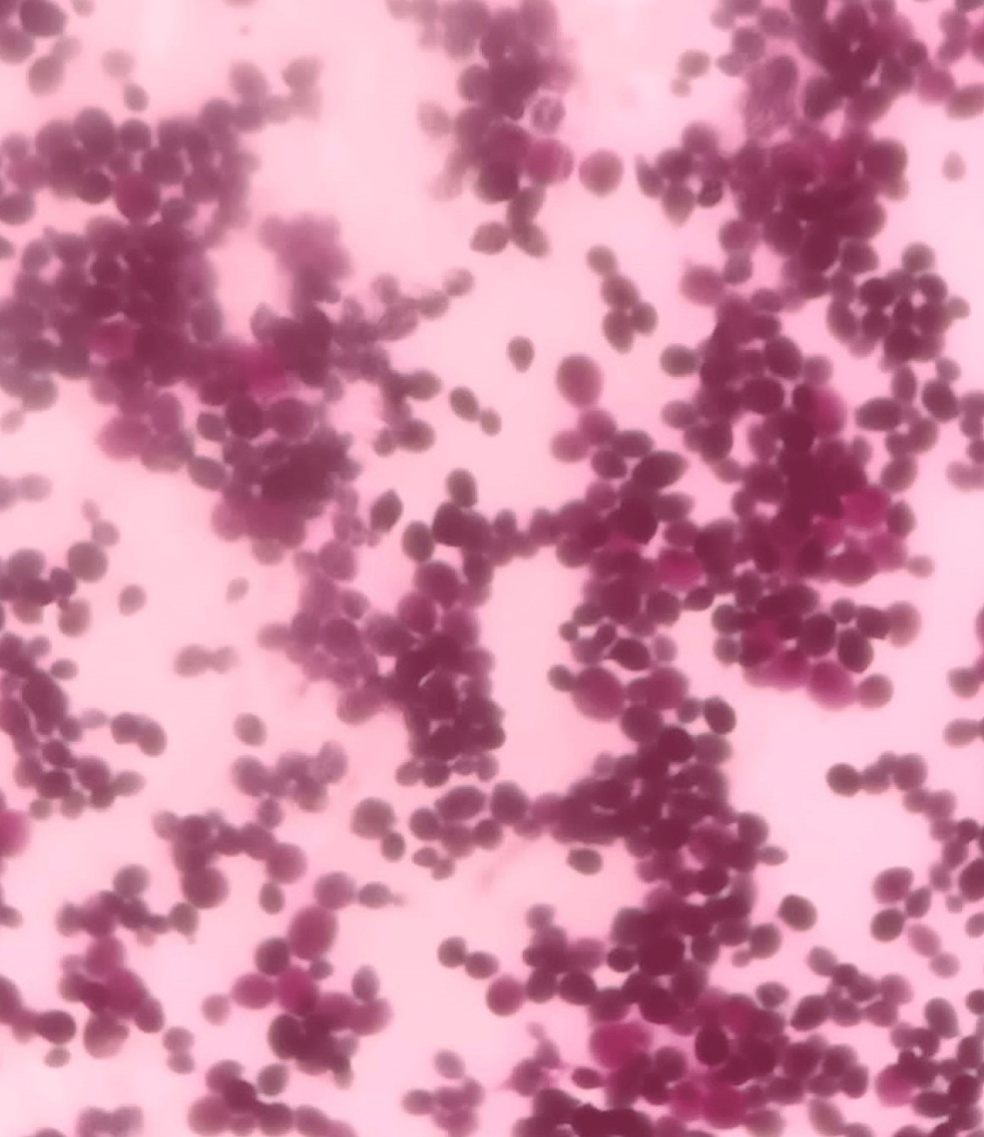
Candida growth in blood culture

An echocardiogram revealed a concentric left ventricular hypertrophy, a normal left ventricular systolic function, and a 23x5 mm linear echo dense mass attached to the atrial side of the anterior tricuspid leaflet with independent mobility (Figure 1). Blood cultures grew methicillin-resistant *Staphylococcus aureus* sensitive to vancomycin and *Candida albicans* (Figure 2) sensitive to fluconazole and liposomal amphotericin B for which he was started on parenteral vancomycin and liposomal amphotericin B at renal adjusted doses. 

**Figure 3:**
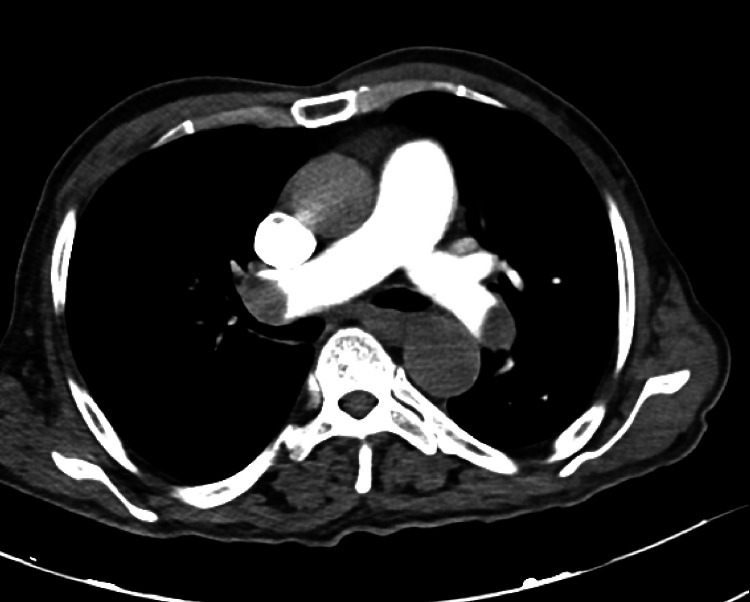
CT pulmonary angiography revealing thrombus

On day 5 of his hospital stay, he developed massive hemoptysis. CT of the chest was done which showed multiple foci of ground-glass opacities which was suggestive of emboli probably of septic origin. Later, a CT pulmonary angiogram (CTPA) was done which revealed a thrombus in pulmonary arteries (Figure 3).

In view of persistent renal dysfunction, pancytopenia, and hemoptysis, an additional workup was done. Serology was negative for hepatitis B, hepatitis C, and human immunodeficiency virus. Auto-immune workup was negative for antinuclear antibodies (ANA), rheumatoid factor (RF), and antiphospholipid antibodies (APLA). The workup was significant for low complement levels and positive perinuclear anti-neutrophil cytoplasmic antibody (p-ANCA) levels. A kidney biopsy couldn't be done because of persistent low platelets. As repeat cultures were also positive, he was planned for lead extraction and device removal with tricuspid valve replacement. However, due to personal reasons, the patient had to leave the hospital against medical advice and was lost to follow-up since then. 

## Discussion

IRGN is an infection mediated by immune complexes with three primary mechanisms: passive entrapment and deposition of circulating complexes, in situ immune complex formation, and in situ complement activation due to the deposition of pathogenic antigens that trigger the infection. This condition is most commonly observed in children, while post-streptococcal glomerulonephritis (PSGN) is most prevalent among individuals aged 2-14 years. In contrast to childhood PSGN and epidemic PSGN, which typically resolve completely with antibiotics, IRGN has a guarded prognosis in adults. Staphylococcal infections have become as common as streptococcal infections among adults. Infections in adults can affect more diverse sites, including the skin, upper respiratory tract, lungs, heart, oral mucosa/teeth, and urinary tract [4]. Clinical presentations differ between children and adults with IRGN. Children typically exhibit nephritic syndrome and hematuria, while nearly 20% of adults with IRGN display nephrotic syndrome, and up to 80% progress rapidly to renal failure. The prognosis for adults is also worse compared to children.

According to Nasr et al. [4], the diagnostic criteria for IRGN are met when at least three of the following are present: (1) presence of infectious disease before or at the onset of GN, (2) hypocomplementemia, (3) endocapillary proliferative and exudative GN on optical microscopy of renal biopsy tissue, (4) C3-dominant deposits on immunofluorescence, and (5) presence of hump-like subepithelial deposits on electron microscopy. IE-associated GN is a condition triggered by infections of the heart valves. This can result from either acute infections, such as in drug abusers or immunocompromised individuals, or subacute infections linked to underlying valvular heart diseases. The histopathology of GN associated with IE presents a focal, segmental, or diffuse proliferative pattern, characterized by endocapillary proliferation and occasional infiltrating leukocytes. However, most of the studies on this subject have been based on autopsy findings in patients with IE-related GN. In a recent study conducted by Boils et al. [5], it was discovered that crescentic GN is the most common finding, with over 50% of patients showing extensive crescent formation and the majority displaying a diffuse rather than a focal pattern of injury. The direct injury and plasmin activation without immune complex deposition are attributed to streptococcal and staphylococcal antigens. Another potential mechanism could be the presence of ANCA antibodies, with up to 28% of cases exhibiting them, as reported in a recent study [6]. Bacterial infections predictably lead to ANCA-positive serology, including cases of suppurative lung disease, and infections caused by *Pseudomonas*, *Klebsiella*, and *E. coli*. The predominant causative agent of IE-associated GN is *Staphylococcus* [7]. In terms of clinical presentation, acute renal failure was the most common, occurring in 79% of IE-associated GN cases, followed by acute nephritic syndrome in 9%, rapidly progressive GN in 6%, and nephritic syndrome in 6% of cases [5].

Diagnosing the condition is typically straightforward when patients exhibit typical signs, symptoms, and serological findings. However, in cases where there is oliguria lasting for more than one week, azotemia persisting for over two weeks, or a progressively deteriorating renal function, many medical centers recommend conducting a renal biopsy to establish a definitive diagnosis. The optimal approach to managing IRGN is a topic of ongoing debate. While antibiotics and addressing the source of infection are the primary treatment methods, some experts propose a trial of steroids due to the immune-based nature of its pathogenesis. Nevertheless, a recent randomized controlled trial conducted at our institution [8] did not demonstrate any significant benefits from steroid treatment. The prognosis for renal failure in IE-IRGN is generally unfavorable. In a case series of 83 patients with GN linked to *Staphylococcus* infection, as reported by Wang et al., approximately 50% experienced persistent renal dysfunction, and 19 patients progressed to end-stage renal disease [9]. Factors that increase the risk of a poor prognosis include older age, diabetes mellitus, pre-existing renal dysfunction before the onset of IE, and the presence of glomerulosclerosis and interstitial fibrosis, as observed through biopsy findings [10].

Infections related to CIED encompass two categories: superficial incisional infections and deep pocket infections. CIED-IE is an increasingly observed occurrence, with an incidence ranging from 0.1% to 5.1%. The most common pathogens responsible for CIED-IE are staphylococci and other Gram-positive bacteria, while more than a third of blood cultures yield negative results. Notably, about 30% of CIED-IE patients experience recurrent pulmonary infections or pulmonary embolisms [11]. Managing CIED-IE presents a challenge for healthcare professionals. Superficial incisional infections affect only the skin and subcutaneous tissues without reaching the pocket. Vigilant patient monitoring is essential to detect early recurrences, which may signal a significant pocket infection. In cases of pocket infection confined to the generator pocket, the appearance of pocket deformations, adherence, or potential erosion often indicates a low-grade, slow-developing infection. If the generator or proximal leads become exposed, the device should be regarded as infected, regardless of culture reports. Pocket infections can be associated with lead infections, CIED systemic infections, and/or IE. Immediate surgical removal is warranted for such pocket infections. Due to the multiple complexities involved in its management, studies have reported mortality rates as high as 35% [12].

Diagnosing CIED-IE involves considering both major and minor criteria, which encompass clinical features, imaging findings, and microbiological investigations. Laguno et al. [13] identified several risk factors associated with the development of CIED-IE, including patients with diabetes mellitus, malignancy, those receiving anticoagulants, individuals on immunosuppressive therapy, and cases involving hematoma in the region around the device insertion site. Potential management strategies for CIED-IE include either exclusive antibiotic treatment or a combination of antibiotics along with the removal of electrical leads, with or without the device itself. However, there is a notable lack of studies comparing these different treatment approaches. While medical management has, on occasion, resulted in a cure, it is associated with high rates of serious and long-term complications, including the exacerbation of CIED-IE-related bacteremia, pericarditis, and bronchopleural fistulas. Recent guidelines dictate that the choice of treatment strategy should depend on the nature of the infection, with superficial infections often managed with antibiotics and deep-seated infections necessitating the removal of the device [14].

## Conclusions

IRGN in adults has a grave prognosis. Hence, it is necessary to identify the inciting antigen and prompt treatment at the earliest. CIED-IE is a disease entity on the rise. Hence, close monitoring and prompt treatment of infections in a patient with any cardiovascular implant can help prevent serious complications. Our case report is one among the rare presentations of IRGN due to CIED-IE. 
